# Atypical Presentation of B-Cell Lymphoblastic Lymphoma: Solitary Scalp Mass in a Pediatric Patient

**DOI:** 10.7759/cureus.67131

**Published:** 2024-08-18

**Authors:** Kristen E Fox, Dwight Philip, Monique Motta, M Ali Ansari-Lari, Tamar L Levene

**Affiliations:** 1 Medicine, Herbert Wertheim College of Medicine, Miami, USA; 2 Pediatrics, Joe DiMaggio Children's Hospital, Hollywood, USA; 3 Pathology, Pathology Consultants of South Broward, Hollywood, USA; 4 Pediatrics, Joe DiMaggio Children’s Hospital, Hollywood, USA

**Keywords:** lymphadenopathy, scalp mass, atypical presentation, pediatric hematology-oncology, b-cell lymphoma, surgical case reports

## Abstract

B-cell lymphoblastic lymphoma (B-LBL) is a subtype of non-Hodgkin lymphoma characterized by the proliferation of abnormal B-cell lymphoblasts in lymphoid tissues. Typical presentations include lymphadenopathy, mediastinal mass, and involvement of organs such as the liver and spleen, but extranodal sites can also be affected. A previously healthy 20-month-old male child presented to the pediatric surgery clinic with a two-month history of a painless, progressively enlarging mass on the scalp as well as postauricular mass consistent with an enlarged lymph node. Ultrasound of the mass near the vertex demonstrated a hypoechoic complex cystic lesion for which excision was indicated. Preoperatively, acute enlargement of the entire postauricular lymphatic chain was noted. Intraoperatively, the scalp mass was noted to be firm with calcified tissue and no identifiable cystic or infectious components. The mass and part of the overlying skin were excised. Pathologic evaluation was consistent with B-LBL. The patient was therefore referred to a pediatric oncologist for further evaluation and management. Bone marrow examination revealed greater than 25% blasts in the clot section, consistent with B-ALL. He was promptly initiated on induction therapy with maintenance chemotherapy to ensure continued remission. This case highlights the atypical presentation of B-cell lymphoblastic leukemia/lymphoma (B-ALL/LBL) as a scalp mass in a 20-month-old male. It underscores the importance of considering malignancy in the differential diagnosis of unusual masses. Prompt collaboration between pediatric surgeons and oncologists facilitates timely diagnosis and initiation of appropriate treatments for optimal outcomes.

## Introduction

Lymphopoietic cancers, including leukemias and lymphomas, account for 40% of childhood cancers in patients under 15 years of age [1,2]. Lymphomas are broadly categorized into two main groups as follows: Hodgkin lymphoma (HL) and non-Hodgkin lymphoma (NHL). In children, NHL accounts for approximately 30% of lymphoblastic lymphomas and the vast majority are of B-cell origin [3]. B-cell lymphoblastic lymphoma (B-LBL) is characterized by the proliferation of abnormal B-lymphocytes and typically presents with enlarging, non-tender lymphadenopathy, symptoms due to compression by a mediastinal mass, or hepatomegaly and/or splenomegaly.

Lymphoblastic lymphoma typically affects people in their late teens and early 20s and is less frequent among young children. Patients most typically present with lymphadenopathy and may have involvement of spleen or liver. Oftentimes, lymphadenopathy may progress rapidly and may be accompanied by respiratory compromise secondary to a mediastinal mass, laboratory abnormalities from bone marrow involvement, or spine or brain involvement. Atypical presentations may involve extra-nodal tissue, such as in this case, though a rapidly enlarging firm scalp mass that followed a remote history of a fall onto the head in an otherwise healthy child has not previously been described. Atypical presentations, although rare, can involve extra-nodal sites such as the central nervous system, bone, skin, or the gastrointestinal tract [4-6]. These cases pose diagnostic challenges, potentially leading to delayed diagnosis and treatment initiation.

In this study, we present a case of a 20-month-old male child with B-LBL presenting as two scalp masses and discuss findings, treatment, and outcomes of postoperative management along with a review of the current literature.

## Case presentation

A previously healthy 20-month-old male child presented to the outpatient pediatric surgery office for evaluation of two painless, progressively enlarging masses located on the scalp (one right posterior auricular and other frontal vertex). The patient’s father stated that these masses had been present for one to two months and denied any recent infections, trauma, or secondary symptoms such as fevers, weight loss, or night sweats. There was a remote history of a fall off of a bed onto the floor at which time the child may have hit his head; however, it was unclear whether the masses were noted before or after the fall.

**Figure 1:**
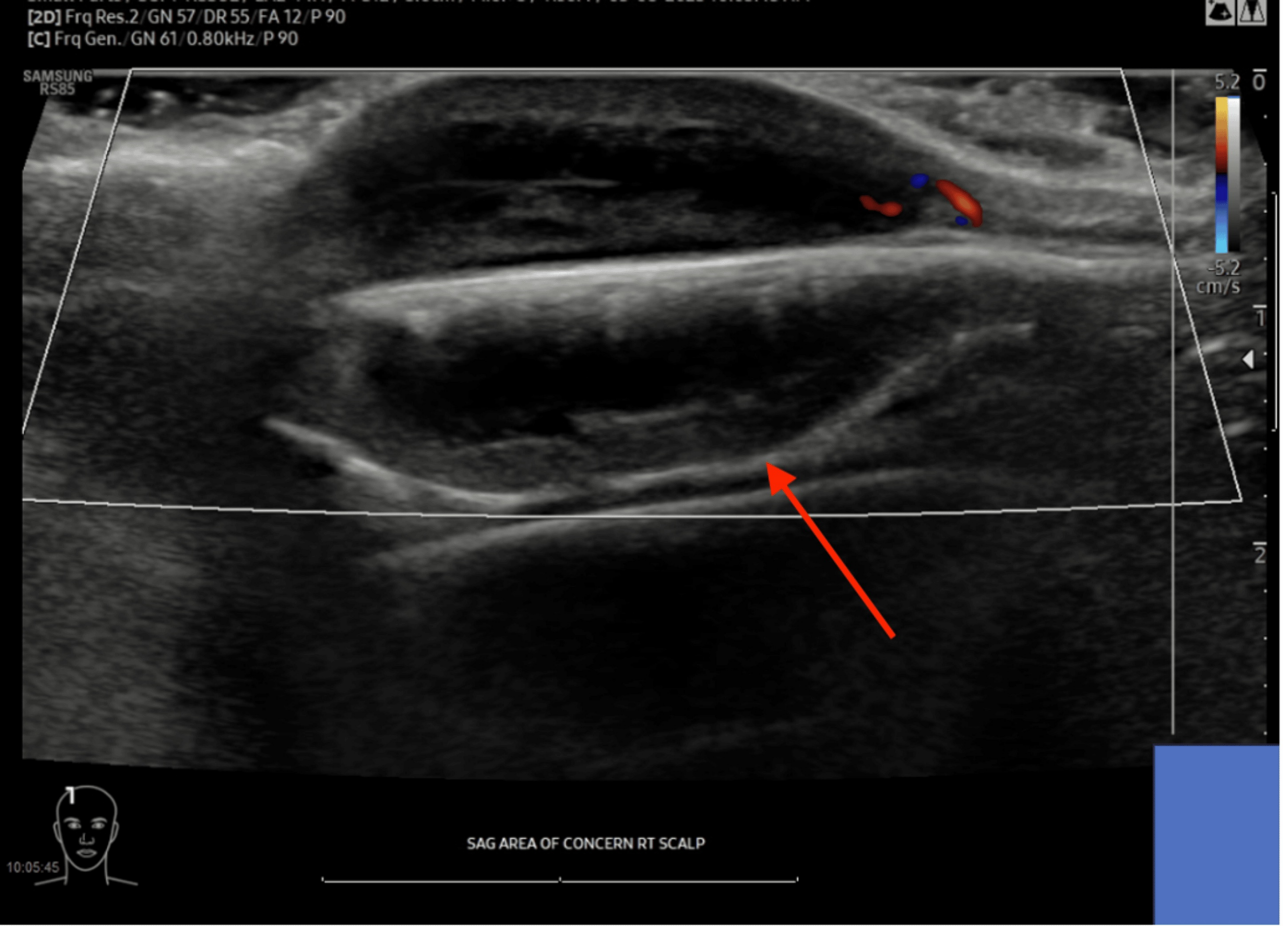
Ultrasound of right frontal mass (arrow), measuring 2.5 cm in length, 2.6 cm in width, and 0.8 cm in depth.

Physical examination revealed two soft tissue masses. The right frontal mass was a firm, non-tender, non-fluctuant swelling on the top of the head to the right of the midline with no overlying hair measuring approximately 3.0 x 3.0 cm. The right postauricular mass was a firm 1.5 x 1.5 cm, well-circumscribed, mobile mass behind the right ear with no overlying skin changes, most consistent with an enlarged lymph node. Ultrasound of both masses was obtained to better characterize the masses and to evaluate for underlying structural involvement. Results of the ultrasound demonstrated that the right frontal mass was consistent with a hypoechoic complex cystic lesion measuring 2.5 x 0.8 x 2.6 cm without deep extension (Figure 1). A cluster of nonenlarged lymph nodes was noted posterior to the right ear measuring 1.0 x 0.4 x 1.0 cm. At this time, mass excision of the right frontal mass was recommended.

As such, the patient was taken to the operating room the following week for a planned excisional biopsy of the right frontal mass. In the preoperative area, the posterior auricular mass was noted to be enlarged compared to the initial evaluation, and additional lymph nodes were palpable in the right cervical region, at levels II and III. An incision was made onto the skin overlying the scalp mass and bilateral skin flaps were created to allow access to the skull circumferentially. On inspection, the scalp mass was noted to be firm with calcified tissue and no identifiable cystic or infectious components. The mass and overlying skin excised from the scalp, revealing that the underlying skull bone was intact and without defects. The excised mass was then sent to pathology for further evaluation. The surgical wound was primarily reapproximated and dressed with triple antibiotic ointment. No operative complications were noted, and the patient was discharged postoperatively on the same day to follow-up pending pathology results.

**Figure 2:**
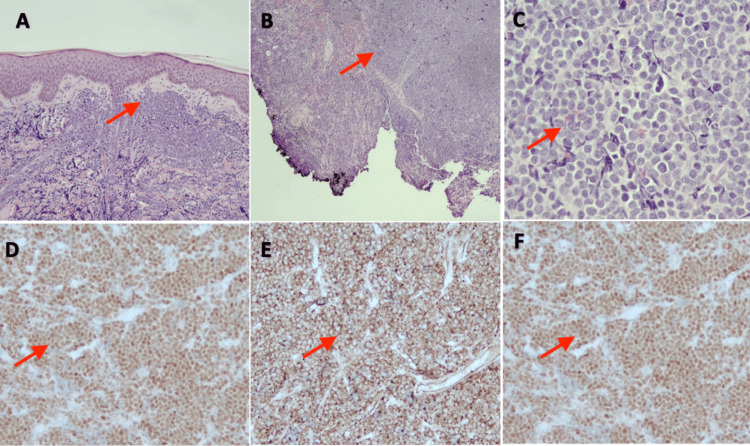
Diffuse mononuclear cell infiltrates in dermis (A) and subcutaneous tissue (B). Blasts with high nuclear-cytoplasmic ratio, scant cytoplasm, and open chromatin pattern (C). Immunohistochemical staining was positive for PAX5 (D), CD34 (E), and TdT (F).

**Figure 3:**
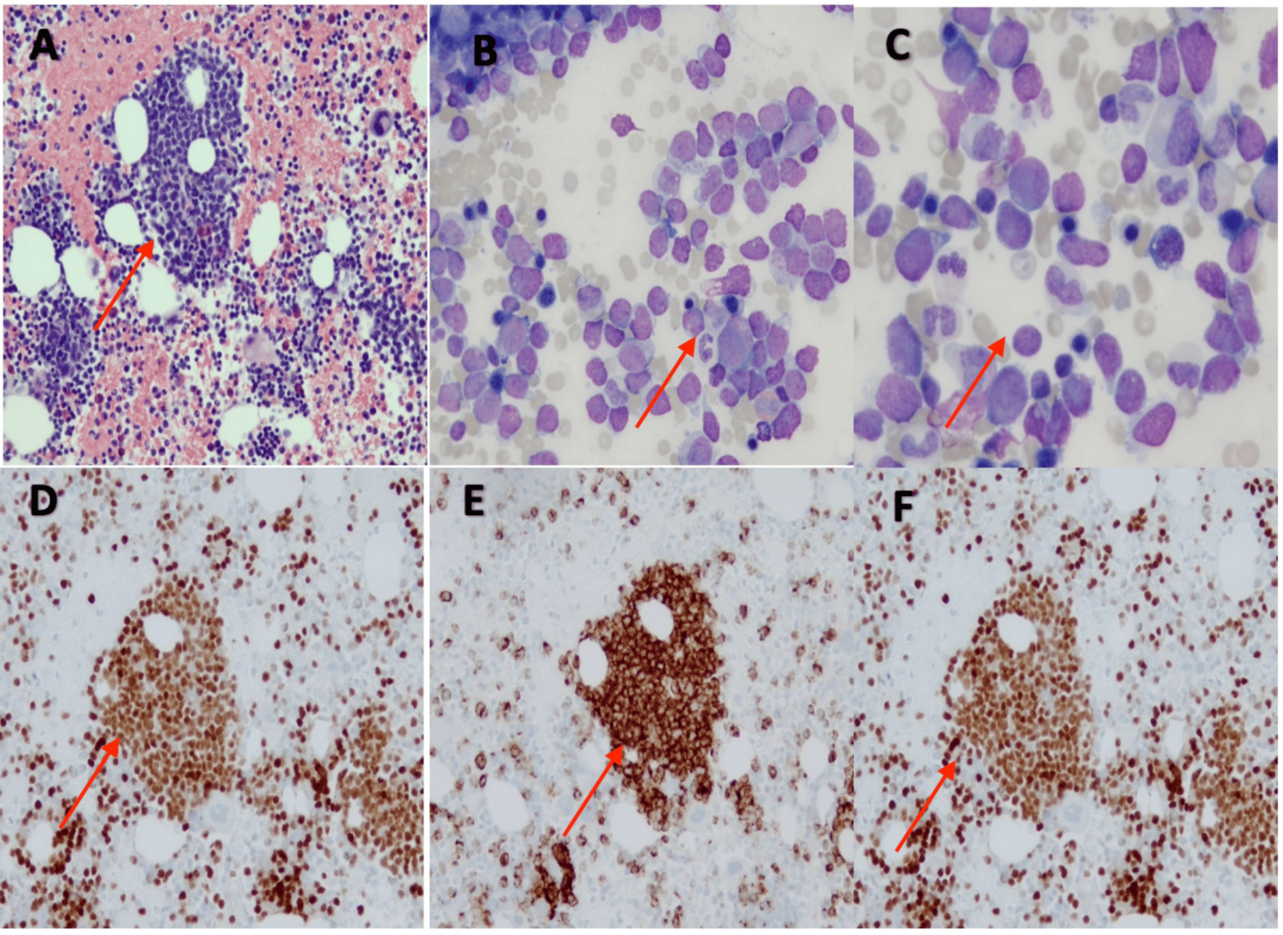
Right posterior iliac crest bone marrow shows immature mononuclear cell aggregates (A). Blasts with high nuclear-to-cytoplasmic ratio and open chromatin (B, C). Immunohistochemical stain was positive for PAX5 (D), CD34 (E), and TdT (F).

Results from the pathology report two days later were consistent with B-cell lymphoblastic lymphoma (B-LBL), with blasts showing a high nuclear-cytoplasmic ratio, scant cytoplasm, and open chromatin pattern. Immunohistochemical staining showed blasts positive for PAX5, CD34, and TdT (Figures 2A-2F). The patient underwent further evaluation by the pediatric oncology service the following week and oncology service management included baseline blood work (CBC, sediment rate, lactate dehydrogenase level, basic metabolic panel, coagulation profile), chest radiograph (x-ray), as well as bone marrow evaluation. Bone marrow evaluation revealed greater than 25% blasts in the clot section, consistent with B-cell acute lymphoblastic leukemia (B-LBL) (Figures 3A-3F). Intrathecal cytarabine, 30 mg, was initiated with diagnostic bone marrow aspirates obtained. A port was placed under general anesthesia six days later, and the patient was initiated on induction therapy per AALL1731 protocol and remained stable throughout the hospital course [7]. He subsequently achieved remission about eight months later. Regular follow-ups with pediatric oncology and maintenance chemotherapy with methotrexate 10 mg every seven days, mercaptopurine 38 mg every night with Bactrim for prophylaxes, were planned to ensure continued remission.

## Discussion

Here, we describe a patient with an atypical subcutaneous presentation of B-LBL. B-LBL is rare and makes up less than 10% of malignant lymphoid cases [8]. It tends to involve children and young adults with a few cases reported in adults older than 65 years old [9]. The highest prevalence is in children under 15 years, estimated at 3.6 per 100,000, with a male-to-female ratio of 1.4 [10]. Typically, extracutaneous manifestations of B-LBL tend to present with erythematous to violaceous lesions overlying the mass with no constitutional symptoms (fever, chills, weight loss) reported at the time of presentation [6,8]. The prognosis for this age group tends to be quite favorable, with a reported five-year survival rate of 86% in children aged 0-14 years [11]. From our review of the literature, we describe a rare presentation of B-LBL where there are no overlying cutaneous changes with well-defined cystic components noted on ultrasound.

According to the fifth edition of the WHO Classification of Hematolymphoid Tumors, the term leukemia (B-ALL) is used when peripheral blood and bone marrow are the primary sites of involvement, and the term lymphoma (B-LBL) when the primary involvement is lymph nodes or extra-nodal sites. The distinction becomes arbitrary when both marrow and non-marrow sites are involved, although many treatment protocols use >25% marrow blasts to define leukemia. Histopathology of B-LBL typically is described as medium-sized lymphoblasts with round, oval, or convoluted nuclei and scant cytoplasm. There is typically an abundance of mitoses and apoptotic cells that would create the classical “starry-sky” pattern, though this pattern is less common in cutaneous lesions of B-LBL [12]. As the histopathologic findings are not specific to B-LBL, ancillary tests like immunohistochemistry or flow cytometry are necessary to establish a definitive diagnosis and exclude other morphologically similar entities, such as T-cell lymphoblastic lymphoma and blastic plasmacytoid dendritic cell neoplasm. Moreover, peripheral blood and bone marrow examination is required to evaluate for the possibility of B-acute lymphoblastic leukemia (B-ALL). The scalp mass revealed uniform positive staining for PAX5, CD79a, CD10, CD34, CD99, TdT, BCL2, and INI consistent with B-LBL. Bone marrow examination revealed greater than 25% blasts in the clot section, with similar immunophenotypic findings, consistent with B-LBL.

The AALL1731 protocol our patient was started on is a phase III clinical trial. The trial tests the addition of blinatumomab to "standard" chemotherapy for patients with newly diagnosed B-cell lymphoblastic leukemia and localized B-LBL. Chemotherapeutic agents typically a part of this protocol can include vincristine, dexamethasone, methotrexate, leucovorin, doxorubicin, cytarabine, mercaptopurine, cyclophosphamide, and thioguanine [7,13]. Blinatumomab is a bispecific T-cell engager antibody construct that simultaneously binds CD3-positive cytotoxic T-cells and CD19-positive B-cells, enabling host T-cells to eliminate CD19-positive B-cells and blasts. It was FDA-approved in 2014, showing improved overall survival in adults with B-cell acute lymphoblastic leukemia [14]. While FDA-approved for pediatric populations, clinical trials are still ongoing to evaluate blinatumomab both in combination with standard chemotherapeutic agents and alone [15]. Our patient remains in remission and stable, still receiving chemotherapy per protocol AALL1731 at 10-month follow-up.

## Conclusions

This case of a 20-month-old male child with B-cell lymphoblastic leukemia/lymphoma (B-ALL/LBL) presenting as a scalp mass emphasizes the importance of considering atypical presentations of pediatric malignancies. Increased awareness of such atypical presentations is necessary for achieving timely diagnosis and optimizing treatment. This case also highlights the pivotal role that multidisciplinary collaboration plays in the prognosis of pediatric oncology patients.

## References

[1] Asselin BL (2020). Epidemiology of Childhood and Adolescent Cancer. Nelson Textbook of Pediatrics. Twenty-First Edition.

[2] Kaatsch P (2010). Epidemiology of childhood cancer. Cancer Treat Rev.

[3] Bassan R, Maino E, Cortelazzo S (2016). Lymphoblastic lymphoma: an updated review on biology, diagnosis, and treatment. Eur J Haematol.

[4] Ducassou S, Ferlay C, Bergeron C (2011). Clinical presentation, evolution, and prognosis of precursor B-cell lymphoblastic lymphoma in trials LMT96, EORTC 58881, and EORTC 58951. Br J Haematol.

[5] Montazer F, Motlagh AS, Dastgir R (2022). Primary cutaneous B-cell lymphoblastic lymphoma presenting with solitary scalp mass in a female child: a case report and review of the literature. Clin Case Rep.

[6] Kim JY, Kim YC, Lee ES (2006). Precursor B-cell lymphoblastic lymphoma involving the skin. J Cutan Pathol.

[7] A study to investigate blinatumomab in combination with chemotherapy in patients with newly diagnosed B-lymphoblastic leukemia. https://www.cancer.gov/research/participate/clinical-trials-search/v?id=NCI-2019-02187&r=1.

[8] Lin P, Jones D, Dorfman DM, Medeiros LJ (2000). Precursor B-cell lymphoblastic lymphoma: a predominantly extranodal tumor with low propensity for leukemic involvement. Am J Surg Pathol.

[9] Chimenti S, Fink-Puches R, Peris K, Pescarmona E, Pütz B, Kerl H, Cerroni L (1999). Cutaneous involvement in lymphoblastic lymphoma. J Cutan Pathol.

[10] Cortelazzo S, Ferreri A, Hoelzer D, Ponzoni M (2017). Lymphoblastic lymphoma. Crit Rev Oncol Hematol.

[11] Luca DC (2021). Update on lymphoblastic leukemia/lymphoma. Clin Lab Med.

[12] Cerroni L (2020). Cutaneous lymphoblastic lymphomas. Skin Lymphoma.

[13] A study to determine the outcomes of patients with localized B cell lymphoblastic lymphoma (B-LLy) when treated with standard risk B-ALL therapy. https://www.mayo.edu/research/clinical-trials/cls-20491489.

[14] Kantarjian H, Stein A, Gökbuget N (2017). Blinatumomab versus chemotherapy for advanced acute lymphoblastic leukemia. N Engl J Med.

[15] Queudeville M, Ebinger M (2021). Blinatumomab in pediatric acute lymphoblastic leukemia - from salvage to first line therapy (a systematic review). J Clin Med.

